# Optimization of acquisition phase and acquisition time window of coronary artery CT angiography with different heart rates based on prospective ECG-gating

**DOI:** 10.1186/s12880-025-01966-w

**Published:** 2025-10-15

**Authors:** Zi-yan Liu, Wei Ding, Ze-peng Ma, Tian-le Zhang, Yong-Xia Zhao

**Affiliations:** 1 Department of Radiology, Affiliated Hospital of Hebei University, Hebei University, Baoding City, Hebei Province 071000 People’s Republic of China; 2 Department of Radiology, Beijing Shijitan Hospital, Capital Medical University, Beijing, People’s Republic of China

**Keywords:** Optimal acquisition phase, Optimal acquisition time window, Coronary computed tomography angiography, Radiation dose, Image quality

## Abstract

**Objective:**

To determine the optimal reconstruction phase and acquisition time window of coronary computed tomography angiography (CCTA) in patients with different heart rates based on prospective ECG-gating and to compare the image quality and radiation dose between the whole cardiac cycle mode and optimized acquisition time window.

**Materials and methods:**

One thousand patients(536male, mean age ± standard deviation, 57.43 ± 11.73years) who underwent CCTA were divided into two groups, group A (*n* = 500)and group B(*n* = 500). According to the heart rate at the time of CCTA, the subjects were divided into subgroups A1 and B1 (< 51 bpm), A2 and B2 subgroups (51–55 bpm), A3 and B3 subgroups (56–60 bpm), A4 and B4 subgroups (61–65 bpm), A5 and B5 subgroups (66–70 bpm), A6 and B6 subgroups (71–75 bpm), A7 and B7 subgroups (76–80 bpm), and A8 and B8 subgroups (81–85 bpm), A9 and B9 subgroups (> 85 bpm). Group A individuals underwent CCTA within a single cardiac cycle and the optimal reconstruction phase at each heart rate was identified based on image quality. The ideal acquisition time window was determined by considering the 95% confidence interval of the best reconstruction phase, which was equivalent to the average value of the best reconstruction phase (standard deviation × 2). The individuals in group B were examined within the optimal collection time window. Compare the radiation doses and image quality of patients in groups A and B.

**Results:**

The findings indicated that the A1-A9 subgroups’ optimal reconstruction phase and acquisition time window were: 61%~85% RR interval; 68%~84% RR interval;70%~82% RR interval and 34%~46% RR interval; 70%~82%RR interval, and 34%~46% RR interval;70%~82% RR interval and 36%~48% RR interval; 65%~89% RR interval and 38%~50% RR interval;68%~84% RR interval and 36%~56%RR interval; 38%~54% RR interval; 38%~58% RR interval. No statistically significant difference was observed in terms of Signal-to-Noise Ratio(SNR), and Contrast-to-Noise Ratio(CNR) between group A and group B, (*P* > 0.05). The average effective radiation dose(ED) values in Group B were 42.6%, lower than in Group A, (*P* < 0.001).

**Conclusions:**

Ideal acquisition phase and acquisition-time windows vary among individuals with varying heart rate(HR). Narrowing the acquisition timeframe based on prospective electrocardiogram(ECG)-gating can considerably lower the radiation dose of CCTA imaging while maintaining image quality.

Coronary artery disease (CAD) continues to have a high death rate, causing a worldwide burden on economies and humanity [1]. CCTA has emerged as the premier option for clinical diagnosis and noninvasive assessment of CAD [2, 3]. However, there has also been an increased focus on concerns regarding excessive ionizing radiation in CCTA [4].

Various approaches are currently employed for reducing the radiation dose in CCTA, such as prospective ECG gating, individualized tube voltage and current management, iterative reconstruction and β-blockers [2, 3, 5]. Compared with the retrospective ECG gating technique, the prospective ECG gating technique can significantly reduce the radiation dose [6, 7]. Simultaneously, the HR influences the quality of CCTA images [8]. Therefore, knowing the law of the optimal collection period of the coronary artery with heart rate is essential for optimizing the collection time window and lowering the radiation dose of CCTA. This study aimed to determine the optimal CCTA reconstruction phase for various HR and optimize the acquisition time window. The image quality and radiation dose of the cardiac cycle and optimized acquisition time window modes were simultaneously compared.

## Materials and methods

### Study population

All research procedures were approved by the Ethics Committee of the Affiliated Hospital, and all patients signed an informed consent form for inspection. The study was conducted in compliance with the Declaration of Helsinkiand and institutional regulations.One thousand patients who underwent CCTA with clinical suspicion of coronary artery disease between January 2023 and November 2023 were prospectively collected, aged from 30 to 87 years (mean age 57.43 ± 11.73years), HR range 37–117 bpm (Table 1). None of the patients received sublingual nitroglycerin.Using the method of random number table, all the subjects were divided into group A (*n* = 500) and group B (*n* = 500).There were no significant differences (*P* > 0.05) in age, sex, height, or weight between groups A and B.According to the HR during CCTA, the subjects were divided into subgroups A1 and B1 (< 51 bpm), A2 and B2 subgroups (51–55 bpm), A3 and B3 subgroups (56–60 bpm), A4 and B4 subgroups (61–65 bpm), A5 and B5 subgroups (66–70 bpm), A6 and B6 subgroups (71–75 bpm), A7 and B7 subgroups (76–80 bpm), and A8 and B8 subgroups (81–85 bpm), A9 and B9 subgroups (> 85 bpm). The HR subgroups (< 51 bpm, 51–55 bpm, 56–60 bpm, etc.) were defined based on a combination of prior literature and clinical relevance [9]. The exclusion criteria were as follows: (1) iodine contrast allergy, (2) heart failure, (3) pregnancy, and (4) metal implants in the scanning field that could affect image quality, (5) Renal insufficiency, (6) arrhythmias (e.g., atrial fibrillation), or significant HR variability (>10 bpm during breath-hold).

**Table 1:** Analysis of general data

	population	Height(cm)	Weight(Kg)	age	gender
Group A	500	166.65 ± 7.95	71.79 ± 12.18	56.49 ± 12.10	269/231
Group B	500	165.44 ± 8.45	70.38 ± 12.97	58.42 ± 11.25	267/233
z/χ2		-1.783	-1.738	-1.941	1.652
*P* value		0.075	0.082	0.052	0.199

### Scanner configuration

All subjects were scanned using a 320-row detector CT(uCT 968,United Imaging) scanner in an advanced supine position with their hands up. We provided the patients with breath-holding instructions before the scan and informed them of matters needing attention throughout the examination. The tube voltage was 100 kVp and the ECG mA modulation was 150 mA-180 mA. The gantry rotation speed was 0.25 s/rot and SFOV 20 × 20 cm. Prospective ECG triggering and axial scan were used in both groups.The scanning range was 120–160 mm and extended from the tracheal Carina to 0.5 cm below the heart base. The reconstruction matrix is 512 × 512 pixels. The acquisition time window in group A was the 0-100% RR interval, indicating the entire cardiac cycle exposure mode. The best reconstruction phase for each subgroup was identified based on the objective evaluation and subjective scores of the right coronary artery, left coronary artery anterior descending branch, and circumflex branch. The optimal reconstruction phase was selected as the time point with the highest combined SNR/CNR and subjective score (≥ 3points by two radiologists) [10]. The optimal collection time window was determined by calculating the 95% confidence interval of the best reconstruction phase, which was the average value of the best reconstruction phase (standard deviation×2). Based on their HR following breath-holds, individuals in each subgroup of group B were assessed by CCTA using group A’s optimal acquisition time window.

### Contrast injection protocol

All individuals were injected with an iodine contrast agent(Iodoferol,320 mg I/ml, Jiangsu Hengrui Pharmaceuticals Co., Ltd, China) through the right median elbow vein at a rate of 4.0 ml/s using a high-pressure syringe. The total amount of contrast agent administered was 0.8 ml/kg, followed by a 40 ml saline injection at the same rate. Scanners utilized smart tracking trigger technology to detect a 6 mm^2^ region of interest in the descending aorta at the four-chamber level. After the CT value of the region of Interest (ROI) reached 132HU, scanning was triggered with a delay time of 6 s.

### Reconstruction

After scanning, the original data images were reconstructed with a thickness of 0.5 mm at uOmnispace.CT. The C-SOFT-AA filter function and hybrid iterative reconstruction technique (KARL 3D) were used for reconstruction with an iterative weight of 8. All images were copied to a computer for post-processing, including multiplanar reconstruction (MPR), curved planar reconstruction (CRP), maximal intensity projection (MIP), and volume rendering (VR).

### Assessment of image quality

#### Objective evaluation

We measured and computed the CCTA horizontal axis images with a 0.5 mm layer thickness. The CT values and standard deviations (SD) of the vessels were measured. Position a 100 mm^2^ circular ROI on the center of the ascending aorta, a 30 mm^2^ area ROI on the same level as the pericardial adipose tissue CT values, and a 1 mm^2^–2 mm^2^ round-like ROI on the RCA, LAD, and LCX. Calcified blood vessel walls and artifacts were avoided during measurement. The SNR and CNR were calculated as follows: SNR = mean CT value of all measured vessels/image noise; CNR= (mean CT value of all measured vessels-CT value of pericardial fat) / image noise; and image noise = mean noise value of all measured vessels.

#### Subjective evaluation

According to the 18-segment standard of the SSCT, the image quality of the left anterior descending (LAD), left circumflex (LCX), and right coronary artery (RCA) was evaluated. Two radiologists with more than 10 years of diagnostic experience evaluated images using a double-blind method. The viewer scored the image quality independently and evaluated it using a 4-point system: 1 point, poor image quality, serious artifacts, and could not be diagnosed; 2 points, adequate image quality, coronary artery artifacts were obvious but did not affect the diagnosis; 3 points, good image quality, minor coronary artifacts, did not affect the diagnosis; and 4 points, excellent image quality, no coronary artifacts. Finally, the average scores of the two physicians were calculated. The subjective score of the image ≥ 3 is considered to meet the requirements of clinical diagnosis [11].

### Radiation dose

All subjects’ volume CT dose index (CTDIvol) and dose length product (DLP) were recorded. The effective radiation dose (ED), ED = DLP × K, was calculated, where K represents the weight coefficient of the X-ray sensitivity of different parts of the heart of patients of different ages, which was 0.014 mSv/mGy cm for the adult heart [12].

### Statistical analysis

The statistical analysis of the data was conducted using SPSS 26.0 software, with the continuous variables being reported as mean ± standard deviation (mean ± SD). The Mann-Whitney U test was employed to compare the ages, heights, and weights of the two groups. Statistical differences in the sex makeup between the two groups were assessed using the chi-square test. Single-factor analysis of variance was used to compare the subjective scores of the two groups and their DLP, ED, SNR, and CNR. The Kappa test was used to assess consistency between observers.

## Results

### Optimal reconstruction phase

**Fig. 1:**
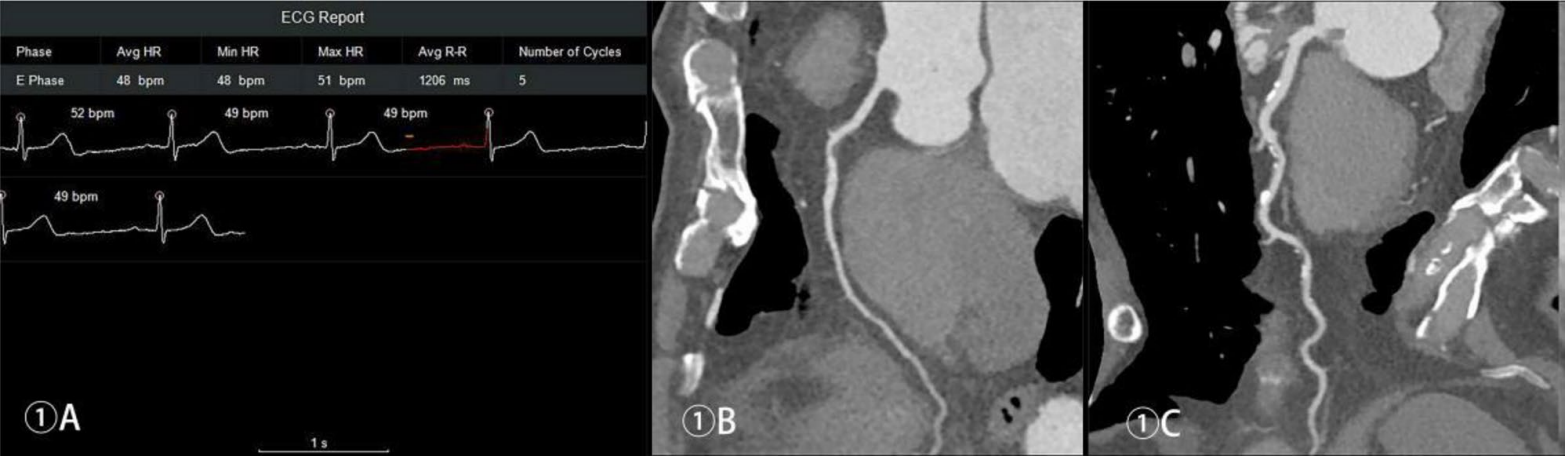
Male, 70 years old, scan window at 61%-85% RR interval, mean subjective image quality score of 4, effective dose (ED) 0.20 mSv

**Fig. 2:**
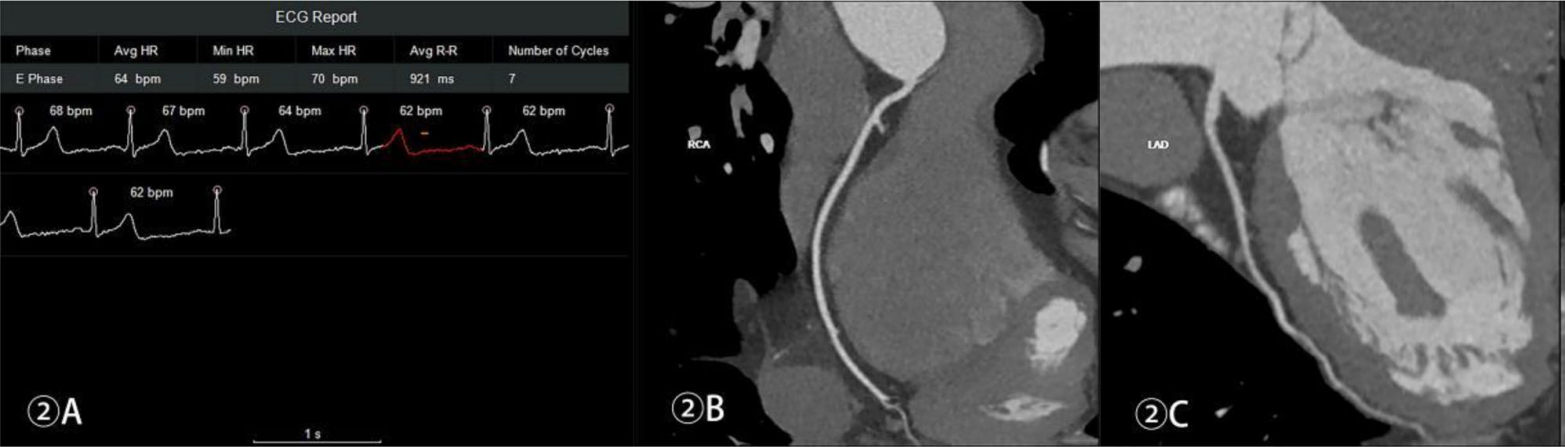
Male, 52 years old, can windows at 34%–46% and 70%–82% RR intervals, mean subjective score of 4, ED 0.26 mSv

**Fig. 3:**
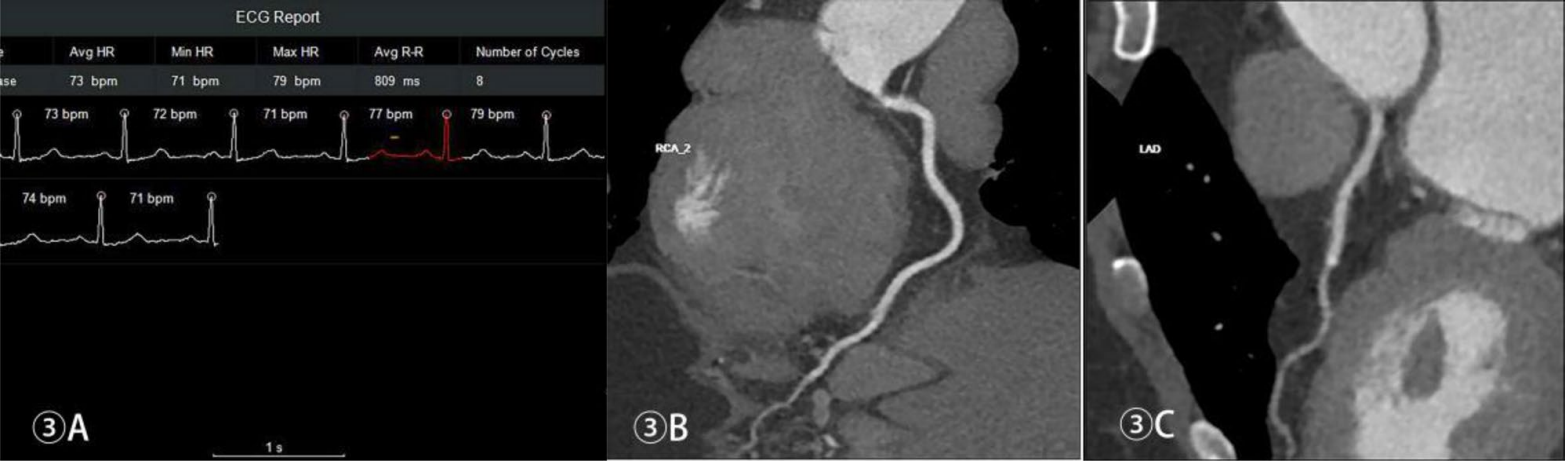
Female, 59 years old, scan windows at 36%-56% and 68%-84% RR intervals, mean subjective score of 3.5, ED 0.25 mSv

**Fig. 4:**
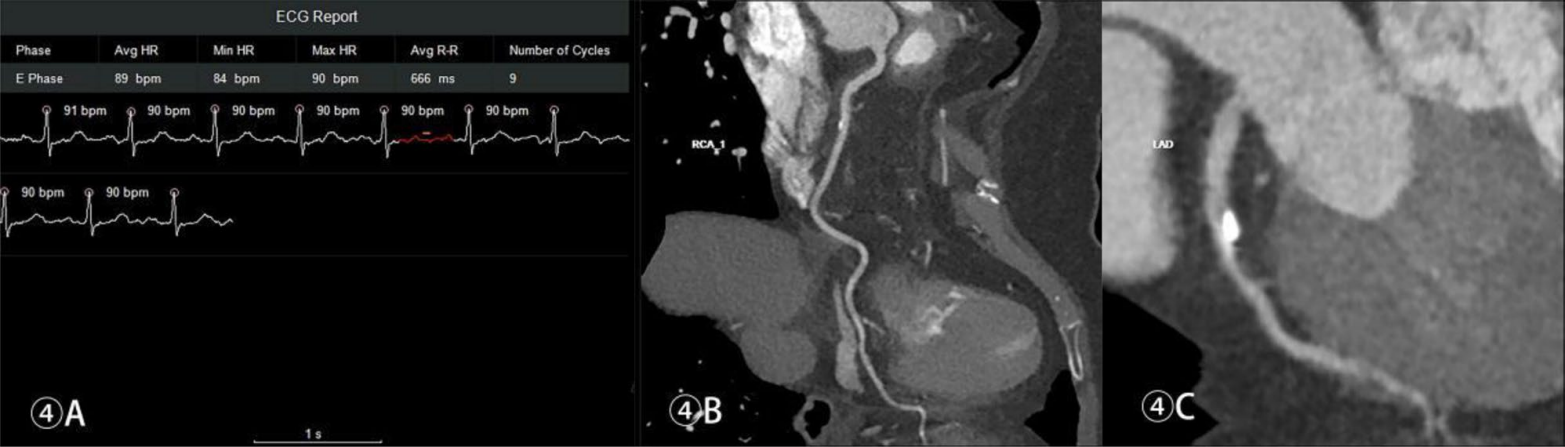
Female, 72 years old, scan window at 38%-58% RR interval, mean subjective score of 3, ED 0.14 mSv

Table 2 shows the optimal reconstruction and collection time windows of the CCTA for each group. Figures 1, 2, 3 and 4 show the acquisition phase diagrams and MPR images of patients with four different heart rates.

**Table 2:** Optimal reconstruction phase and acquisition time window of CCTA for subgroups in group A

HR(bpm)	Optimal reconstruction phase(%RR interval)	acquisition time window(% RR interval)
<51	73 ± 6	61–85
51–55	76 ± 4	68–84
56–60*	76 ± 3/40 ± 3	70–82 34–46
61–65*	76 ± 3/40 ± 3	70–82 34–46
66–70*	76 ± 3/42 ± 3	70–82 36–48
71–75*	77 ± 6/44 ± 3	65–89 38–50
76–80*	46 ± 5/76 ± 4	36–56 68–84
81–85	46 ± 4	38–54
>85	48 ± 5	38–58

*:It indicates that this cohort utilized a dual-phase acquisition strategy

Figures 1, 2, 3 and 4. Four cases of different heart rates using the optimized acquisition window.

### Assessment of image quality

#### Objective evaluation

Fig. 3 and 4 show the mean values of CT, standard deviation, SNR, and CNR for the LAD, LCX, RCA, and aorta in groups A and B. The CT value, SD, SNR, and CNR did not differ significantly between the two groups. There was no significant difference in the subjective scores of the three blood vessels (*P* > 0.05). The two radiologists had good consistency(*P <* 0.05), the K values of the right coronary artery, left anterior descending artery, and circumflex artery were 0.88, 0.89, and 0.89 respectively.

#### Subjective evaluation

**Fig. 5:**
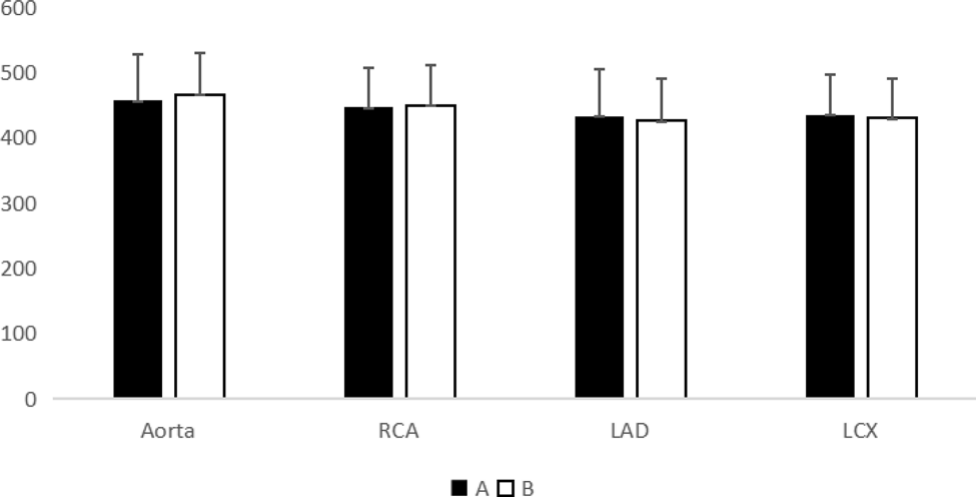
CT value of group A and B

**Fig. 6:**
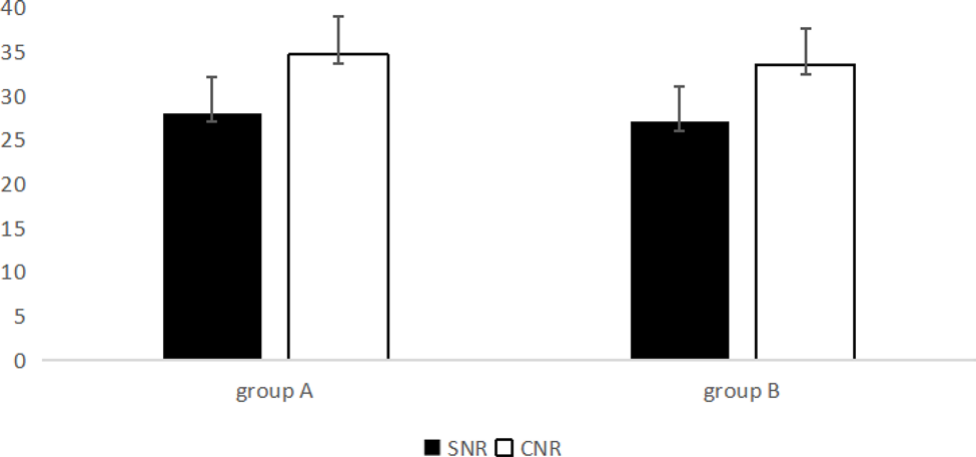
SNR CNR of group A and B

The subjective scores of the two radiologists for the CCTA images of the two groups are shown in Figs. 5 and 6. Statistical analysis revealed no significant difference in the subjective scores of the CCTA images between the two groups.

### Radiation dose

Table 3 displays the mean CTDIvol, DLP, and ED values for patients in groups A and B. According to the statistical analysis, CTDIvol, DLP, and ED were all lower in group B than in group A (*P* < 0.001). In group B, the average CTDIvol, DLP, and ED decreased by 42.7%, 42.6%, and 42.6%, respectively. Table 4 shows the mean ED values for the subgroups in groups A and B. The findings demonstrated a statistically significant difference (*P* < 0.05) in the mean ED value between groups A and B.

**Table 3:** Radiation doses to subjects in groups A and B

	DLP(mGy·cm)	CTDIvol(mGy)	ED(mSv)
Group A	418.19 ± 59.41	30.211 ± 3.891	5.85 ± 0.83
Group B	239.97 ± 45.05	17.315 ± 3.14	3.36 ± 0.63
*F* value	1689.002	1971.015	1689.002
*P* value	*P* < 0.001	*P* < 0.001	*P* < 0.001

**Table 4:** Radiation doses for subgroups of groups A and B

HR(bpm)	ED mean(mSv)		ED reduction	Population	
	Group A	Group B		Group A	Group B
<51	7.29 ± 0.69	3.40 ± 0.76	53.36%	37	43
51–55	6.61 ± 0.44	3.67 ± 0.66	44.48%	73	70
56–60	6.10 ± 0.45	3.64 ± 0.46	40.33%	67	71
61–65	5.64 ± 0.39	3.53 ± 0.20	37.41%	85	89
66–70	5.51 ± 0.67	3.33 ± 0.45	39.56%	70	63
71–75	5.33 ± 0.73	3.15 ± 0.44	39.42%	49	54
76–80	4.94 ± 0.46	2.91 ± 0.46	41.09%	44	40
81–85	4.69 ± 0.37	2.47 ± 0.54	47.33%	41	38
>85	4.30 ± 0.54	2.04 ± 0.32	52.56%	34	32

## Discussion

CCTA is maturing with the ongoing advancements in CT technology, and its clinical application has become increasingly widespread. It is currently used as the primary method for evaluating coronary heart disease [13–15]. However, high radiation doses are drawing increasing attention from the public.

The HR of the subjects is one of the key factors for the success of CCTA [16]. Prospective electrocardiographic gating technology only acquires data when the heart is moving relatively slowly. The acquisition time window is narrowed compared with the common full cardiac cycle mode, and the exposure dose is reduced or even non-exposed at times outside of the acquisition time window; therefore, it has become one of the effective means of reducing the radiation dose of CCTA [17]. It has been demonstrated that the optimal imaging time of the coronary arteries is located in mid-to-late diastole in subjects with low HR, in late systole in those with high HR, and in late systole or mid- to late-diastole in those with a mid-range HR [16, 18, 19]. The results of the present study were partially inconsistent with these findings, with 12.5% of the subjects in subgroup A3 (56–60 bpm) having optimal reconstruction phases in systole and 29.4% of the subjects in subgroup A7 (76–80 bpm) having optimal reconstruction phases in diastole. This may be explained by the fact that the subjects in subgroup A3 had a slower HR and longer systolic period; therefore, there were time points when the heart moved more slowly in systole.

The results of this study also showed that the optimal reconstruction phase gradually shifted backward with an increasing HR. This is consistent with the results of tissue Doppler waveform regression analysis used to predict the optimal phase of the coronary arteries [20]. As the HR increased, the diastole was significantly shorter than the systole, the relative resting time of diastole was significantly shorter, and the optimal reconstruction phase shifted from diastole to systole. According to the data analysis in this study, 80 bpm is the critical HR for the diastolic-to-systolic reconstruction of CCTA images. Simultaneously, the acceleration of HR increases the temporal resolution requirement of the CT scanner. At HR of > 100 bpm, the temporal resolution required for imaging was 150 ms. This explains the higher CCTA failure rate in patients with high HR. In this study, optimizing the acquisition time window significantly reduced the radiation dose. However, through objective (no significant difference in SNR and CNR) and subjective (scores ≥ 3, meeting clinical diagnostic requirements) evaluations, it was confirmed that diagnostic accuracy was not affected. For the subgroup with mid-range HR (such as 76–80 bpm), the dual-phase acquisition strategy further ensured the stability of image quality and avoided the risk of missed diagnosis due to an overly narrow time window.

Full cardiac cycles or wider acquisition time windows are often used to ensure the success rate and image quality of CCTA [7, 15]. This led to a significant increase in the radiation dose to the examinees. The results showed no statistically significant differences in image quality between the two groups. However, the mean radiation dose in Group B was significantly lower than in Group A. The mean ED in group B decreased by 42.6% compared to in group A. This was due to the narrowing of the acquisition time window in group B and the shortening of the time the patients received exposure. The reduction in radiation dose was particularly obvious in subgroups B1, B2 (< 55 bpm), B8, and B9 (> 80 bpm), which decreased by 53.4%, 44.5%, 47.3%, and 52.6%, respectively, compared with subgroups A1, A2, A8, and A9. This is because the above four groups were optimized with only one acquisition time window. In contrast, all other groups had two acquisition time windows and still received low-dose X-rays for exposure between the two acquisition windows. A study found that the automatic coronary-specific reconstruction technique (Smart Phase) provides image quality comparable to expert manual selection in high HR patients, while significantly improving workflow efficiency by saving 60% of time, benefiting less experienced users [21].

The limitations of this study are as follows: (1) The sample sizes of the subgroups were not the same. The sample size of the subgroups with higher HR was smaller, and the sample size of the higher HR should be increased during follow-up, (2) Due to the influence of temporal resolution, there were fewer subjects with a HR of > 100 bpm in this study, and whether the conclusions of the present study apply to subjects with a higher HR needs to be confirmed by further research, and (3) This study relies on a single CT model of a single institution, which limits the universality of the conclusions. (4) The optimized acquisition time window proposed in this study is only applicable to patients with sinus rhythm and stable HR (fluctuation ≤ 10 bpm). For patients with arrhythmias or those requiring beta-blockers, this strategy may not ensure image quality.

## Conclusion

Based on our findings, we recommend the integration of narrow acquisition windows into standard CCTA workflows for patients.Patients with different heart rates (HR) have distinct optimal acquisition phases and time windows: for low HR (≤ 55 bpm), the scanning window narrows to mid-to-end diastolic phase (HR < 51 bpm: 61–85% RR interval; HR 51–55 bpm: 68–84% RR interval); for mid-range HR (56–80 bpm), it narrows to end-systolic or mid-to-end diastolic phase (HR 56–65 bpm: 70–82 and 34–46% RR interval; HR 66–70 bpm:70–82 and 36–48% RR interval; HR 71–75 bpm: 65–89 and 38–50% RR interval; HR 76–80 bpm: 36–56 and 68–84% RR interval); for high HR (≥ 81 bpm), it narrows to end-systolic phase (HR 81–85 bpm: 38–54% RR interval; HR > 85 bpm:38–58% RR interval). Reasonable optimization of the acquisition time window via a wide-body detector can significantly reduce CCTA radiation dose in patients with stable HR while ensuring image quality.

## Data Availability

The datasets used for this study is not publicly available due to protect study participants privacy but is available from the corresponding author on reasonable request.
